# Comment on “Using Innovative Acoustic Analysis to Predict the Postoperative Outcomes of Unilateral Vocal Fold Paralysis”

**DOI:** 10.1155/2018/6269593

**Published:** 2018-03-15

**Authors:** Andrea Lovato, Cosimo de Filippis

**Affiliations:** Department of Neuroscience, University of Padova, Audiology Unit at Treviso Hospital, Treviso, Italy

We read with interest the study by Tsou et al. [1] comparing an innovative acoustic measure, that is, the number of irregular peaks (NIrrP), with jitter and shimmer as measured by the Multi-Dimensional Voice Program (MDVP); the authors wanted to verify the ability of these three acoustic measures to predict the surgical outcome of fat injection laryngoplasty in unilateral vocal fold paralysis. Using the receiver operating characteristic (ROC) curve, they found that NIrrP [Area Under the ROC Curve (AUC) = 0.98, 95% confidence interval (CI) = 0.78–0.99] was a more accurate predictor of long-term surgical outcome than jitter (AUC = 0.73, 95% CI = 0.47–0.91) and shimmer (AUC = 0.63, 95% CI = 0.37–0.85) [1].

AUC is frequently used to test the ability of clinical variables to predict the outcome in various clinical settings, especially to identify patients at a higher risk of carcinoma recurrence [2–4]. Furthermore, the efficacy of the discrimination could be measured with the scale proposed by Hosmer and Lemeshow [5, 6]. This scale is used to evaluate the discriminatory power of statistical models as follows: AUC equal to 0.5, no discrimination; AUC between 0.7 and 0.8, acceptable discrimination; AUC between 0.8 and 0.9, excellent discrimination; AUC beyond 0.90, outstanding discrimination [5, 7].

In 2016, we used the Hosmer and Lemeshow scale to compare the discriminatory power of two pieces of voice analysis software, MDVP and Praat, in distinguishing the gender of euphonic adults [8]. We found no discrimination for shimmer in dB with either MDVP (AUC = 0.658) or Praat (AUC = 0.682); on the other hand, MDVP absolute jitter achieved an acceptable discrimination between males and females (AUC = 0.752), and Praat absolute jitter achieved an outstanding discrimination (AUC = 0.901) [8]. Using the same scale on Tsou et al.'s results [1], we could say that the innovative acoustic measure NIrrP showed outstanding discriminatory power (AUC = 0.98) in identifying unilateral vocal fold paralysis patients at a higher risk of surgical failure after fat injection laryngoplasty. Research on this and other predictors of surgical failure in phoniatry should be encouraged.
